# Clinical and Preclinical Advances in Gastroenteropancreatic Neuroendocrine Tumor Therapy

**DOI:** 10.3389/fendo.2017.00341

**Published:** 2017-12-04

**Authors:** Judy S. Crabtree

**Affiliations:** ^1^Department of Genetics, Louisiana State University Health Sciences Center, New Orleans, LA, United States

**Keywords:** neuroendocrine tumor, Notch, small intestinal NET, pancreatic neuroendocrine tumor, carcinoid

## Abstract

The molecular events leading to gastroenteropancreatic neuroendocrine tumor (GEP-NET) formation are largely unknown. Over the past decades, systemic chemotherapies have been replaced by therapies directed at particular molecular targets such as the somatostatin receptors, mTOR complexes or proangiogenic molecules. These approaches have demonstrated some success in subtypes of this heterogeneous tumor group, but responses are still widely varied. This review highlights the clinical trials ongoing for neuroendocrine tumors (NETs) and includes emerging immunotherapy, which holds great promise for NETs based on successes in other tumor types. Current avenues of preclinical research, including Notch and PI3K/AKT, will lead to additional targeted therapies based on genome-wide studies that have cast a wide net in the search for driver mutations. Future preclinical and clinical investigations are required to identify those mutations predictive of therapeutic response or disease progression. Results of current clinical trials outlined here will better inform patient management with respect to agent selection, timing, duration and combination therapy in the treatment of NETs.

## Introduction

Gastroenteropancreatic neuroendocrine tumors (GEP-NETs) are clinically and biologically heterogeneous, resulting in widely varied clinical outcomes and challenges when establishing guidelines for tumor characterization and patient management. Over the past decade, significant effort has been expended to standardize the classification system used for these tumors in the clinical setting (1, 2). In 2010, guidelines published by the World Health Organization (WHO) recommended categorization of GEP-NETs based on clinical behavior, histopathological features and proliferation rate (3). These guidelines defined “neuroendocrine” as neoplastic cells that express markers of neural lineage (synaptophysin and chromogranin A). The term “neuroendocrine neoplasm (NEN)” was adopted which includes all tumors or carcinoma that derives from neuroendocrine cells. This led to the establishment of three morphologically distinct groups: (1) well-differentiated NENs, (2) poorly differentiated NENs [also called neuroendocrine carcinoma (NECs)], and (3) mixed adenoneurocarcinoma (MANECs). Well-differentiated NENs were further subdivided based on proliferative activity into either G1 (≤2% Ki67 index and mitoses <2/10 high-power field) or G2 (3–20% Ki67 index and mitoses 2–20/10 high-power field). All poorly differentiated NECs are G3 (>20% Ki67 index and >20/10 high-power field). MANECs were defined as having >30% of each component in the tumor and were defined as NEC G3 containing non-neuroendocrine components (typically adenocarcinoma) (3).

In recent months, the WHO2017 grading system was published which modifies the original WHO2010 classification for pancreatic NETs in several important ways (4). First, clinical practice noted a group of tumors that was discordant with their grade based on established criteria—meaning tumors that exhibited Ki67 indices of >20% even though they were well differentiated. Despite this discordance, these tumors were often classified as G3, yet behaved clinically as well-differentiated G1/2 tumors in that they had poor responses to platinum-based chemotherapy, surgery resulted in favorable outcomes, and these tumors responded well to somatostatin analogs, mTOR inhibitors, and alkylating agents such as temozolomide (5–7). This resulted in the generation of a new category called panNET G3 to account for these tumors. Second, the Ki67 index cutoff value was raised to 3% instead of 2% for pNET G1/2 distinction. Third, the MANEC classification was too narrow in that it assumed that the non-neuroendocrine component was glandular in nature, when in fact it can also be well-differentiated tumor such as pNET G1/2. Therefore, the term “mixed neuroendocrine non-neuroendocrine neoplasm” (MiNEN) was adopted (8). The WHO2017 grading recommendations are found in Table 1.

**Table 1:** WHO2017 grading criteria for pancreatic neuroendocrine neoplasms (NENs).

	panNENs			panNECs
Grade	G1	G2	G3	G3
Ki67 index	<3%	3–20%	>20%	>20%
Mitotic index	<2/10 hpf	2–20/10 hpf	>20/10 hpf	>20/10 hpf

**Immunohistochemistry**				
P53	Weak <20%	Weak <20%	Weak <20%	Strong
RB1 loss	−	−	−	Strong
Islet 1	+	+	+	−
SSTR2A	+	+	+	−^a^
DAXX/ATRX loss	−	−	−	+

*hpf, high power fields*.

*^a^In 80% of panNECs*.

Staging for NETs has undergone repeated revision by groups in the US and Europe in attempt to gain consensus within the field and to accurately develop informative, prognostic, and biological classifications to aid in patient management, study comparisons, and to direct future clinical trial design (9, 10). A recent study of pancreatic NETs proposed amended guidelines that are a fusion of two of the most widely used staging criteria: the American Joint Commission on Cancer (AJCC; primarily used in North America) and the European Neuroendocrine Tumor Society (ENETS; primarily used in Europe) (11). This study addressed shortcomings in both systems and proposed a modified version, mENETs that uses the standard TNM classification from ENETS while incorporating the AJCC staging definitions (11). This mENETs system was tested on data from the SEER database and found to be more appropriate than either system alone for pancreatic NETs (11). In addition, guidelines for the surgical management of small bowel NETs were released recently by the North American Neuroendocrine Tumor Society (NANETS) (12), as were guidelines for medical management and follow-up surveillance of patients with GEP-NETs (13, 14).

The incidence of GEP-NETs is 2–5 cases per 100,000 and this number is expected to continue rising primarily due to improvements in diagnostic imaging and physician awareness (1, 15–17). Patients with GEP-NETs often present with advanced disease at diagnosis (18). Surgical resection is often the first option either for curative (for localized, non-metastatic disease) or palliative (for advanced metastatic disease) intent, followed by pathway-based, systemic chemotherapies such as those outlined below for patients with metastatic disease.

## Target-Based Therapies—Gastroenteropancreatic NETs (GEP-NETs)

### Somatostatin Analogs

SSAs (octreotide, lanreotide, pasireotide) were initially developed to mimic the inhibitory action of somatostatin on cell surface G-protein-coupled receptors called SSTR1–5, which mediate downstream hormone release and cell growth *via* PI3K and MAPK signaling (19). These drugs are used successfully to control symptoms of the carcinoid syndrome that is clinically characterized by flushing, diarrhea and right-side heart valve disease as a result of hypersecretion of bioactive amines from small intestinal NETs (20). The ELECT trial (NCT00774930), a phase III double-blind study of lanreotide depot as a therapy for carcinoid syndrome, enrolled 115 patients—59 in the lanreotide depot arm and 56 in the placebo arm. Enrolled patients were randomized and given access to short-acting octreotide as a rescue medication, with the primary study endpoint being the utilization of the rescue medication and self-reported frequency of diarrhea and/or flushing episodes. The percentage of days using rescue medication was significantly decreased in the lanreotide depot group (33.7%, 95% CI 25–42.4) compared to placebo (48.5%, 95% CI 39.6–57.4) (21) indicating increased control of carcinoid syndrome with lanreotide.

SSA therapy was also used in a trial of patients with Hashimoto’s thyroiditis and enterochromaffin-like hyperplasia (22). Chronic atrophic gastritis is an autoimmune attack on the parietal cells that results in increased gastrin production. Chronic atrophic gastritis is also a risk factor for gastric endocrine carcinoma, especially when correlated with enterochromaffin-like cell hyperplasia. One study evaluated the prevalence of chronic atrophic gastritis, hypergastrinemia, and enterochromaffin-like cell hyperplasia in patients with Hashimoto’s. Treatment with SSAs caused regression of enterochromaffin-like cell hyperplasia in all patients suggesting that the early diagnosis of enterochromaffin-like cell hyperplasia, and the treatment with SSAs, may play a role in prevention of gastric endocrine carcinoma. However, it should be noted that one patient in this study with a type-1 gastric carcinoid at study start did not have significant tumor regression in response to SSA (22).

In clinical trials focused on NEN regression/control, SSAs have demonstrated efficacy in treatment arms compared to placebo controls. The phase 3 PROMID trial (NCT00171873) examined octreotide LAR (*n* = 42) compared to placebo (*n* = 43) in treatment-naïve, well-differentiated, metastatic midgut (mostly GI) NENs with time to tumor progression (TTP) as the primary endpoint. Octreotide LAR significantly lengthened TTP by 8.3 months over placebo [14.3 months TTP for octreotide LAR, 6 months in placebo (HR = 0.34; 95% CI 0.2–0.59; *p* = 0.000072)] (23). Long-term follow-up of these patients did not show a demonstrable increase in overall survival (OS) (24). Patients on placebo were offered octreotide LAR at study completion and the crossover design of this study may have confounded the data interpretation with respect to OS (23, 24). The CLARINET trial (NCT00353496) included lanreotide depot (*n* = 101) compared to placebo (*n* = 103) in metastatic, non-functioning, low-grade (G1 or G2) pancreatic and intestinal NETs with stable disease (25, 26). This trial resulted in significantly prolonged PFS in the lanreotide depot group compared to placebo (median not reached versus 18 months; HR = 0.47; 95% CI 0.3–0.73; *p* < 0.001). As in the PROMID trial, this improvement in PFS did not translate to increases in OS at 2 years (25). The CLARINET trial was continued for an additional 40 months with an open label extension that further confirmed the favorable safety and tolerability profile of lanreotide depot with long-term use (26). The ongoing phase 2 CLARINET FORTE study (NCT0265987) is investigating a reduced dosing interval (14 versus 28 days) for lanreotide depot in patients with well-differentiated pancreatic or midgut NENs with the primary endpoint of PFS.

Peptide receptor radionuclide therapy (PRRT) has tremendous potential for the treatment of NETs with elevated expression of SSTRs or other cell surface receptors. This therapy directs radionuclides directly to cancer cells by targeting the SSTRs expressed on their surface (27). It should be noted that SSAs have varying affinities for the different SSTR receptors and may be a compounding factor when comparing clinical trials involving different SSAs (28, 29). The NETTER-1 trial (NCT01578239), measured PFS in patients treated with octreotide LAR with and without ^177^Lu-Dotatate as a localized anticancer radiotherapy for advanced, SSTR-positive metastatic midgut (primarily jejuno, ileum, and proximal colon) NETs. In a preliminary analysis published recently, treatment with ^177^Lu-Dotatate plus octreotide LAR (*n* = 111) resulted in markedly increased PFS compared to the octreotide LAR (*n* = 110) alone (30). At the time of the planned interim analysis, the median PFS had not been reached for the ^177^Lu-Dotatate plus octreotide LAR group and was 8.4 months for the control group (95% CI 5.8–9.1). The estimated rate of PFS at month 20 was 65.2% (95% CI 50–76.8%) in the ^177^Lu-Dotatate plus octreotide LAR group versus 10.8% (95% CI 3.5–23) in control. PRRT has also been studied in combination with standard chemotherapeutic agents in NETs. For example, small published studies of ^177^Lu-dotatate in combination with capecitabine to radiosensitize in metastatic GEP-NETs, demonstrated safety and efficacy (31, 32). Other studies examined the combination of ^177^Lu-octreotate with capecitabine and temozolomide in advanced, low-grade NETs (*n* = 30), demonstrating an overall response rate of 80% (95% CI 66–93), with complete remission in 13% (95% CI 4–30) and partial response in 70% (95% CI 52–83) (33, 34). Among the many phase II trials in progress to study PRRT in NETs, the CONTROL NETS trial (NCT02358356) is testing the combination therapy of ^177^Lu-octreotate + capecitabine + temozolomide is compared with capecitabine + temozolomide in low- to intermediate-grade (G1/G2) pancreatic NENs and versus PRRT alone in low- to intermediate-grade midgut NENs. The LUNET trial is recruiting 98 patients for a non-comparison trial of two separate doses of ^177^Lu-Dotatate in advanced enteropancreatic NETs (NCT02489604), and the LUCAS trial (NCT02736448) is a randomized trial seeking to enroll 176 patients to compare ^177^Lu-PRRT + capecitabine versus 177Lu-PRRT alone in well-differentiated GEP-NENs. The PRELUDE trial (NCT02788578) is a retrospective study seeking to describe the effects on PFS of combination Lanreotide depot with PRRT in metastatic well-differentiated (G1 or G2) GEP- and bronchopulmonary NETs. There are many other PRRT trials in NETs currently recruiting (clinicaltrials.gov) and these trials are just the beginning of studies to understand combination interventions in NETs. It is unknown whether the timing and sequence of therapies is important, i.e., simultaneous versus sequential, direct effect versus radiosensitizing, octreotide versus pasireotide versus lanreotide (efficacy depends on the SSTR distribution present in tumor, if any), and many of the current clinical trials will yield informative results in coming years. Guidelines for therapy selection, timing, and combination may also be further clarified as we understand more about the genetics of the different subtypes and grades of NETs. Table 2 outlines the current phase III clinical trials for GEP-NETs.

**Table 2:** Current Phase III clinical trials for gastroenteropancreatic neuroendocrine tumors (GEP-NETs).

NCT Number	Title	Intervention
NCT02288377^a^	A Study Evaluating Lanreotide as Maintenance Therapy in Patients with Non-resectable GEP-NETs (REMINET)	Lanreotide Placebo
NCT02246127	Efficacy and Safety of Everolimus and STZ-5FU Given One Upfront and the Other Upon Progression in Advanced pNET (SEQTOR)	everolimus STZ-5FU
NCT02588170	Phase III Study of Sulfatinib in Treating Advanced Extrapancreatic NET	Sulfatinib Placebo
NCT02589821	Phase III Study of Sulfatinib in Treating Pancreatic NETs	Sulfatinib placebo
NCT03049189	Efficacy and Safety of ^177^Lu-edotreotide PRRT in GEP-NETs	^177^Lu-edotreotide everolimus
NCT01842165	^177^Lu-octreotate Treatment Protection using Multimodality Imaging in Refractory NETs (LUMEN)	^177^Lu-octreotate
NCT02465112	Metabolic Radiotherapy after Complete Resection of Liver Metastases in Patients with Digestive NET	^111^In-Pentetreotide placebo
NCT01578239	A Study Comparing Treatment with ^177^Lu-DOTA^0^-Tyr^3^-octreotate to Octreotide LAR in Patients with Inoperable, Progressive, SSTR-Positive Midgut Carcinoid Tumors	Octreotide LAR ^177^Lu-DOTA^0^-Tyr^3^-octreotate
NCT02608203^a^	^68^Ga-DOTANOC pET/CT in GEP-NETs	^68^Ga-DOTANOC

*^a^Phase II/III*.

### Gastric Inhibitory Polypeptide Receptor

In addition to SSTR, other peptide receptor targets have also been identified and utilized for *in vivo* scintigraphy and targeted radiotherapy. Incretin receptors, including glucagon-like peptide-1 (GLP-1R) (35, 36) and glucose-dependent insulinotropic polypeptide receptor (GIPR) (37, 38), have been investigated with earnest in recent years, with putative utility in tumors such as insulinoma that often occur with low or absent expression of SSTR. Interestingly, in studies of SSTR-negative gastrointestinal or bronchial NETs, more than 88% were positive for GIPR (39). Recent studies from the Maecke lab (40) investigate a set of GIP-derived ligands for their ability to image a broad spectrum of GIPR-expressing NETs with positive results. Whereas biodistribution studies in mouse xenograft models indicate accumulation of these peptides in the kidney which could result in nephrotoxicity upon translation to clinical use, optimization of GIP-derived peptides demonstrates great potential for both imaging and PRRT in GIPR-positive NETs.

### mTOR Pathway Inhibitors

The mammalian target of rapamycin (mTOR) pathway was initially studied in NETs as a part of familial tumor syndromes known to have genetic mutations in genes upstream of the mTOR complexes. For example, the autosomal-dominant syndromes neurofibromatosis type 1 (NF1) and tuberous sclerosis (TS) are caused by inactivating mutations in *NF-1* and *TSC1/2*, respectively. Patients with NF1 develop a spectrum of NETs in the ampulla of Vater, duodenum, and mediastinum, and demonstrate constitutive activation of the mTOR signaling pathway as a result of *NF1* loss (41). TS has been associated with pancreatic NETs, and the loss of the TSC1/2 genes similarly leads to activation of mTOR signaling (42). Clinical genetic studies have identified that gastrointestinal (small intestine) NETs can have somatic mutations in *MEN1, CDKN1B*, and other genes involved in the PI3K/AKT/mTOR signaling pathway (43–45) and more recent whole-exome sequencing data have identified that 14% of pancreatic neuroendocrine tumors (NETs) have mutations in genes of the mTOR signaling pathway (46).

Mammalian target of rapamycin functions as an intracellular serine/threonine kinase that modulates key cellular processes such as nutrient sensing, proliferation, metabolism, and cell survival. mTOR exists as one of two known complexes, mTORC1 or mTORC2, in conjunction with several other proteins (47). Inhibitors of mTOR (rapamycin, everolimus/RAD001, temsirolimus) bind to the FK506 binding protein, which then binds to mTORC1 and inhibits pathway signaling. Everolimus has been investigated in NETs through the RADIANT series of trials. The RADIANT-1 trial was an open-label, phase 2 study that initially investigated everolimus in patients with advanced, low- to intermediate-grade GEP-NENs and included a small number of patients with carcinoids (*n* = 30) and islet cell tumors (*n* = 30). Patients received everolimus either alone or in combination with octreotide at the discretion of the study investigators and resulted in promising antitumor activity that was well tolerated (48). Octreotide is known to reduce serum IGF-1 levels, a pathway upstream of the mTOR pathway, so the combination of everolimus plus octreotide was considered a two-pronged approach to accomplish both upstream and downstream inhibition of mTOR signaling. Patients receiving the combination therapy had median PFS of 60 weeks (95% CI 54–66), compared to 50 weeks with 5 mg everolimus alone (95% CI 23–78) and 72 weeks with 10 mg everolimus alone (95% CI 60–83). However, this study was not powered nor designed to make the comparison between these study groups. RADIANT-1 was followed up with a larger study, the RADIANT-2 trial (NCT00412061) which was a randomized, double-blind, placebo-controlled phase III study (49). Patients with low- or intermediate-grade NENs (the majority small intestinal primary site) were treated with everolimus plus octreotide LAR (*n* = 216), or placebo plus octreotide LAR (*n* = 213). Everolimus plus octreotide LAR improved PFS by 5.1 months over the everolimus plus placebo group (16.4 months, 95% CI 13.7–21.2 versus 11.3, 95% CI 8.4–14.6), but did not meet statistical significance. Treatment with everolimus offered patients a 23% decrease in relative risk of progression (HR 0.77; *p* = 0.026) compared to placebo (49). The recently completed COOPERATE-2 trial, a randomized, open-label phase II study had similar results (50). Patients with advanced, well-differentiated progressive pancreatic NETs were treated with either everolimus alone (*n* = 81) or in combination with pasireotide (*n* = 79) and evaluated on the primary endpoint of PFS. The everolimus alone group had a median PFS of 16.6 months, while the combination group had median PFS of 16.8 months (HR 0.99; 95% CI 0.64–1.54), however, the combination group showed an advantage in partial responses with 20.3 versus 6.2% in the everolimus alone group (50), suggesting that there may be some low-level benefit to combination therapy.

The RADIANT-3 trial (NCT00510068) extended the study of everolimus alone to a larger number of patients with advanced, low, or intermediate grade pancreatic NETs with radiologic evidence of progression within the prior 12 months. Patients in this international, multicenter, double-blind phase 3 trial were randomized to receive everolimus (*n* = 207) or placebo (*n* = 203) in addition to supportive care (which involved SSA therapy in 40% of patients across both groups). Median PFS in the everolimus group was 11 months (95% CI 8.4–13.9) compared to the placebo group at 4.6 months (95% CI 3.1–5.4) and offered a reduction in relative risk of progression of 65% (HR = 0.35; 95% CI 0.27–0.45; *p* < 0.001) (51). The RADIANT-4 trial (NCT01524783) further studied everolimus in patients with advanced, progressive, well-differentiated, non-functional NETs of the lung or gastrointestinal tract (ileum and rectum) (52, 53). This randomized, placebo-controlled phase III trial investigated everolimus (*n* = 205) versus placebo (*n* = 97) on the background of best supportive care. Overall, patients receiving everolimus demonstrated an increase of 7.1 months PFS compared to placebo (11 months, 95% CI 9.2–13.3 versus 3.9 months, 95% CI 3.6–7.4) and a 52% decrease in relative risk of progression or death (52, 53). In the GI subset of these patients (175 of the 302 enrolled), PFS was increased by 7.7 months (13.1 months, 95% CI 9.2–17.2 in everolimus versus 5.4 months, 95% CI 3.6–9.3 in placebo), with no unexpected safety concerns (53). Taken together, the RADIANT-3 and -4 trials demonstrate that mTOR inhibitors provide durable antitumor effects in NETs from pancreatic, lung, and gastrointestinal origin suggesting that treatment with everolimus, with or without SSA therapy, may continue to be a promising treatment option for patients with progressive, advanced stage NETs. Indeed, there is now a phase 4 clinical trial in progress in China (NCT02842749) to understand the long-term safety of everolimus in locally advanced or metastatic, well-differentiated progressive pancreatic NETs.

Everolimus has also been studied in combination with PRRT. The NETTLE phase 1b study conducted in Australia was a proof of concept study for combination of everolimus with ^177^Lu-octreotate to identify the maximum tolerated dose, any dose limiting toxicities and to evaluate objective response in patients with progressive low-grade GEP-NENs (*n* = 16; 11 small bowel, 5 pancreatic). This study reported an overall response rate of 44% (7/16), and all patients maintained stable disease throughout the 6-month course of treatment with a manageable (and apparently reversible) side effect profile (54). Four of five pancreatic NEN patients achieved PR with this combination therapy, which sets the stage for an appropriately powered larger, phase 2 trial for statistical comparisons of combination versus monotherapy on the endpoint of PFS.

### Antiangiogenic Therapies

Neuroendocrine tumors are highly vascular tumors and overexpression of proangiogenic molecules and their receptors such as vascular endothelial growth factor (VEGF)/VEGF receptor (VEGFR), platelet-derived growth factor (PDGF)/PDGFR, fibroblast growth factor (FGF)/FGFR, and epithelial growth factor (EGF)/EGFR have been reported (55, 56). These observations have led to extensive preclinical and clinical investigations using tyrosine kinase inhibitors (TKI; either small molecule antagonists or blocking antibodies) that inhibit the activity of proangiogenic signals (57).

The oral TKI sunitinib (which targets both VEGFR and PDGFR) was studied in a prospective phase III clinical trial in patients with advanced, well-differentiated pancreatic NETs (NCT00428597). This trial resulted in a 6-month increase in PFS (11.4 months in the sunitinib group versus 5.5 months in the placebo arm; HR 0.42; 95% CI 0.26–0.66; *p* < 0.001) (58). This study was discontinued early by the independent ethics review panel because of a high number of serious adverse events and deaths in the placebo arm, and a significant trend toward improvement in PFS in the treatment group (58). Another small molecule TKI sulfatinib (which targets VEGFR and FGFR) has completed phase I studies (NCT02133157), with an objective response rate of 26.5% (9/34) and a disease control rate of 70.6% (24/34) (59). The phase I study identified 300 mg sulfatinib as the recommended dose for a phase Ib/II trial (NCT02267967) that is nearing completion. Randomized, double-blind phase III trials are currently recruiting to test sulfatinib versus placebo in patients with advanced pancreatic NETs (NCT02589821) and with low/intermediate-grade (G1/G2) advanced extrapancreatic NETs (any location except pancreatic; NCT02588170).

Pazopanib is an inhibitor of VEGFRs 1, 2, and 3 and showed clinical activity in phase II trials (NCT01280201, NCT01099540, NCT01841736) as a monotherapy for advanced GEP-NETS with stable disease or confirmed PR in 75.7% of patients (28/37; 95% CI 58.8–88.2) (60, 61). Combination of pazopanib with octreotide depot (NCT00454363) was associated with tumor response in advanced, well-differentiated pancreatic NETs but not in carcinoids (62), suggesting that a larger phase III trial in advanced pancreatic NETs is warranted. Axitinib (inhibits VEGF and PDGF) had disappointing results as a monotherapy in progressive, advanced grade G1/G2 extrapancreatic NETs (NCT01435122). Tumor growth was inhibited in advanced carcinoids, with radiographic evidence of stable disease in 21/30 (70%) patients, but there was an unacceptably high incidence of hypertension associated with this therapy (63). Lenvatinib is another pan-TKI in an ongoing phase II trial (NCT 02678780, TALENT study) evaluating ORR as the primary endpoint in patients with advanced pancreatic NETs after progression on other therapy (arm1) or in progressive gastrointestinal NETs after SSA therapy (arm2).

Bevacizumab is a recombinant human IgG1 monoclonal antibody that blocks VEGF from binding to VEGFR, resulting in decreased blood vessel density around tumors in rectal cancer studies (64). Initial phase II trials evaluating bevacizumab in advanced carcinoids and well-differentiated metastatic NETs were positive with increases in PFS (65, 66). Bevacizumab was studied in combination with temozolomide in patients with advanced NETs (56% carcinoid, 44% pancreatic; NCT00137774). Interestingly, only pancreatic NETs responded to therapy (5/15, 33%) with no responses in carcinoids (0/19, 0%) suggesting that this regimen may be more efficacious in patients with pancreatic NETs (67). More recently, the XELBEVOCT trial (NCT01203306) studied octreotide LAR + capecitabine ± bevacizumab in well to moderately differentiated metastatic NETs (multiple anatomic primary sites). Of the 45 patients enrolled in the study, partial response (most often in pancreatic NETs) was noted in 8 patients (17.8%; 95% CI 6.4–28.2) (68). A subsequent phase II trial followed this to evaluate the combination of octreotide LAR + everolimus ± bevacizumab (NCT01229943) in locally advanced or metastatic pancreatic NETs that cannot be treated by surgery. Surprisingly, preliminary data available on clinicaltrials.org indicate that there is no statistically significant improvement in PFS with the octreotide + everolimus group at 14 months (95% CI 9.1–16.9) versus 16.7 months (95% CI 12.6–19.7) in the octreotide + everolimus + bevacizumab group (*p* = 0.12). The phase III SWOG S0518 study (NCT00569127) investigated octreotide + bevacizumab compared to octreotide + interferon alpha-2b in advanced G1/G2 NETs (various anatomical sites). This study concluded there was no discernible difference in PFS between these two treatment groups, suggesting that bevacizumab had similar activity to that of interferon alpha-2b therapy (69).

### Immunotherapy

Immunotherapy is a recent, rapidly emerging therapeutic option for all cancers, including NETs. Immune checkpoint inhibitors block interactions of programmed death-ligand 1 (PD-L1)/programmed cell death receptor 1 (PD1) or cytotoxic T lymphocyte antigen 4 (CTLA4) to block immune escape of tumor cells. PD-L1 is expressed on the surface of many cancer cells and interacts with its receptor PD1 on the surface of T cells. Immune escape occurs when antigens produced by the tumor inhibit T cells, allowing cancer cells to remain undetected by immune surveillance. Antibodies that target and block PD-L1 (avelumab), PD1 (pembrolizumab, nivolumab, JS001, or PDR001), or CTL4 (ipilimumab) have been used in a number of cancers with promising results, including melanoma, renal, lung, prostate, and bladder cancers, and equivalent trials are now in progress for GEP-NETs. Furthermore, durable responses have been obtained with the anti-PD-L1 antibody avelumab in clinical trials of Merkel Cell carcinoma, a NET of the skin, suggesting successful proof of concept for immune therapy in other NETs. Immunohistochemical analysis of PD-L1 expression in intermediate- to high-grade (G2/G3) GEP-NEN/NECs (*n* = 32, the majority from pancreas and rectum) was measured in 22% of patients and correlated specifically with the aggressive, high grade (G3) tumors. Based on these results, there are a number of clinical trials in progress or soon to open that investigate immunotherapy in GEP-NETs (Table 3).

**Table 3:** Clinical immunotherapy trials in gastroenteropancreatic neuroendocrine tumor (GEP-NETs).

NCT number	Immunotherapy	Tumor type	Phase
NCT03167853	JS001 (humanized anti-PD1 antibody)	Advanced, well-differentiated neuroendocrine tumor (NET) following first line failure	1b

NCT03043664	Pembrolizumab	Non-resectable, recurrent, or metastatic well or moderately differentiated GEP-NET	1/2
	Lanreotide depot		

NCT02923934	Ipilimumab	Pancreatic NET	2
	Nivolumab	Intestinal NET	

NCT02834013	Ipilimumab	Pancreatic NET	2
	Nivolumab	Intestinal NET	

NCT02939651	Pembrolizumab	Metastatic high-grade GEP-NETs who have failed platinum-based therapy	2

NCT02955069	PDR001 (humanized anti-PD1 IgG4 antibody)	Well-differentiated, non-functional GI, pancreatic or thoracic NET	2
		Poorly differentiated GEP-NEC	

## Preclinical Pathways Under Investigation—Enteropancreatic NETs

### Genetic Profiling of GEP-NETs

Despite the abundance of activity in clinical research, the underlying molecular mechanisms of NET tumorigenesis are not fully understood and continue to be actively investigated in the preclinical setting. Recent whole-exome and whole-genome sequencing efforts have begun to reveal the mutational and epigenomic landscape of NET subtypes. The identification of new germline and somatic mutations, along with copy number variations and other changes, has identified novel mechanisms such as Notch, chromatin remodeling, histone modification, and promoter methylation that may be contributing to pathogenesis. These investigations with genetic underpinnings have the potential to be future precision approaches beyond the currently targeted methodologies outlined above.

There have been several reports published on the genetic profiles of different NET subtypes (70, 71). Gebauer et al. identified the degree of genomic instability and identified frequent copy number variations present in low-grade (G1/G2), well-differentiated pancreatic NETs. Using array comparative genomic hybridization, copy number gains were found on 6p22.2-p22.1, 17p13.1, 7p21.3-21.2, and 9q34.11, affecting regulatory genes involved in transcription, signaling, and epigenetic control (70). Recent whole-exome (46) and whole-genome (72) sequencing of pancreatic NETs identified that the most commonly mutated genes in this tumor type are genes involved in chromatin remodeling, such as multiple endocrine neoplasia type 1 (*MEN1*), death domain-associated protein (*DAXX*), α-thalassemia/mental retardation syndrome, X-linked (*ATRX*) (46), and DNA repair genes *MUTYH, CHEK2*, and *BRCA2* (72). Interestingly, these studies demonstrated very little pathogenic role for oncogenes commonly mutated in cancer such as *TP53* or *RB1*. Protein products from *DAXX* and *ATRX* form a heterodimer that is required for chromatin remodeling through histone H3.3 at telomeres. In the Jiao study, clinical NET samples with mutations in these genes were associated with improved prognosis (46). However, follow-up studies by other groups wherein telomere-specific FISH and DAXX/ATRX immunohistochemistry were performed, loss of these gene products was associated with worse prognosis for NET patients (73, 74). Other studies in patients with MEN1-related pancreatic NETs suggest that DAXX/ATRX mutations are not driver mutations, but rather later events in the pathogenesis of NET development (75). Although there is still some controversy over the clinical correlation of *DAXX/ATRX* mutations, this area of research has attracted a great deal of attention. Both the mutations in *MEN1* and *DAXX/ATRX* suggest that targeting these epigenetic mechanisms with small molecule compounds may be a viable approach to developing new therapeutics for pancreatic NETs.

Small intestinal NETs have also been profiled at the genetic level (76–78). The study performed by Banck et al. on 48, G1/G2, well-differentiated SI-NETs identified single-nucleotide variants in a number of known cancer genes, including *FGFR2, MEN1, HOOK3, EZH2, MLF1, VHL, NONO*, and *SMAD1* by whole-exome sequencing. The dysregulated genes implicated several altered cellular processes, such as chromatin remodeling, DNA damage pathways, apoptosis, and RAS signaling. 30% of SI-NETs have genetic mutations in the PI3K/AKT/mTOR pathway (see below) and amplification of AKT1 and 2 occurs the most frequently (44). In a subsequent study, CDKN1B mutations were identified in 8% of tumors, suggesting the gene for p27 to be a putative tumor suppressor in these tumors, with an obvious role in regulating the cell cycle (45, 79). Studies on copy number alterations in SI-NETs identified losses of 11q23.1-qter, 16q12.2-qter, 9pter-p13.2, and 9p13.1-11.2, and gains in 14q11.2, 14q32.2-32.31, 20pter-p11.21, 20q11.1-q11.21, and 20q12-qter (80). These regions impact tumor suppressor genes, growth factors (in particular *FGF2/FGFR3/FGFB, PDGR*), and signal transduction pathways (*TGFB1, IGFBP3, AKT1, E2F1*). DNA methylation studies have provided a global picture of gene regulation, highlighting genes, such as *WIF1, RASSF1A, CTNNB1, CXCL14, NKX2-3*, and others with increased promoter methylation, and at the same time noting a significant decrease in global methylation in tumors compared to normal reference controls (81). Furthermore, Karpathakis et al. performed a large, integrated analysis of these tumors and showed epigenetic-based mutations in 85% of tumors with 21 dysregulated genes, including *CDX1, CELSR3, FBP1*, and *GIPR* (79).

In general, GEP-NETs can be divided by location into gastrointestinal and pancreatic, but also by different copy number aberrations, gene expression profiles, and distinct DNA methylation patterns (82–84). Pancreatic NETs have variations in *DAXX, ATRX, PTEN*, and *TSC2*, whereas GI-NETs have identified *CDKN1B* and *RASSF1A* as the recurrent mutations. Ras-associated domain family 1 (RASSF1) is frequently hypermethylated in pancreatic NETs—75% of 48 well-differentiated tumors demonstrated hypermethylation at this site, with no hypermethylation in adjacent normal tissue (85). Furthermore, this hypermethylation correlated with larger tumors and widespread metastasis. Expression of *O*(6)-methylguanine DNA methyltransferase (MGMT), a DNA repair enzyme that guards cells against mutations caused by O6 alkylating agents, has also been studied in pancreatic NETs (86, 87). Temozolomide cytotoxicity is attributed to its ability to induce DNA methylation at the O6 position of guanine, leading to DNA mismatch and tumor cell death (88). MGMT promoter methylation was reported in 40% of pancreatic NETs (85), suggesting that this might be a biomarker for response to temozolomide. Others demonstrate that MGMT deficiency due to methylation was more prevalent in pancreatic NETs compared to gastrointestinal NETs (89). Subsequent studies have not identified a correlation between MGMT protein expression and promoter methylation, even though MGMT promoter methylation was significantly associated with response to temozolomide (86, 87). All of these genomic investigations have led to ongoing clinical tissue collection protocols to identify biomarkers (NCT02092714, NCT03130205) and complete molecular profiling (NCT02586844) of NET subtypes to aid in diagnosis and patient management.

### Canonical Notch Signaling

Through genome-wide studies, the Notch signaling pathway has been implicated in pathogenesis of GEP-NETs as well. Notch has been studied for many years in the context of cancer; and over the years, the major players in the pathway have been identified, revealing a complex and sometimes redundant signaling network. The Notch pathway is widely recognized as a central player in proliferation, differentiation, and stem cell maintenance. Notch signaling is evolutionarily conserved and canonical signaling relies on the presence of a Notch receptor (in mammals called Notch1–4) binding to a ligand present on a neighboring cell. Ligand binding promotes intracellular cleavage of the receptor by metalloproteases (ADAM and gamma-secretase) to release the active form of the receptor, called the Notch intracellular domain (NICD). The NICD translocates into the nucleus, binds to transcription factor CBF-1/Suppressor of Hairless/LAG-1 (CSL, also known as RBP-Jκ) and activates transcription of Notch-responsive genes (90).

Notch is known to contribute to tumorigenesis in epithelial-derived cancers by inhibiting differentiation, promoting cellular proliferation, and/or inhibiting apoptosis. There is evidence that Notch can behave as a tumor suppressor or oncogene in these cancers depending on the cellular context. In the case of GEP-NETs, few studies have comprehensively examined the complement of Notch molecules and the mechanisms of Notch signaling. Those that have demonstrate that the presence of Notch signaling components varies across cells derived from the neuroendocrine lineage. Immunohistochemical staining for Notch1, Hes1, Hey1, pIGF1R, and FGF2 antibodies on a tissue microarray of 120 well-differentiated NETs arising from the pancreas (*n* = 74), ileum (*n* = 31), and rectum (*n* = 15), demonstrated elevated Notch1 expression in 100% rectal, 34% of pancreatic, and 0% of ileal NETs. Hes1 expression was present in 64% of rectal, 10% of pancreatic, and 0% of ileal NETs (91), exhibiting significant variability in Notch1 signaling across different tissue types. Due to the low/absent expression of Notch in ileal NETs/carcinoids, some have proposed that Notch functions as a tumor suppressor in these tumors. In the non-tumorigenic cell, Notch signaling activates a cascade of events that ultimately leads to the inhibition of a protein called ASCL1 (92). In ileal NETs, ASCL1 is overexpressed and transient overexpression of Notch1 in carcinoid cell lines *in vitro* can reverse ASCL1 overexpression, suggesting that activation of Notch1 may be therapeutic. In addition, organic extracts such as resveratrol activate Notch *in vitro* and have been proposed as therapy in patients with low-grade GI-NETs (NCT01476592 (93, 94)).

The presence of coactivators and corepressors also dictates Notch functionality. CSL coactivators such as MAML, SKIP, and p300 are well known to activate transcription of Notch target genes by binding to NICD, while in the absence of NICD, corepressors such as SMRT (95), SIRT (96), and others (97) inhibit such functions. Notch activator and repressor complexes can also actively remodel the chromatin at Notch-responsive target genes and provide an additional layer of reversible epigenetic regulation (98) by recruiting proteins with histone modification potential (99–101). A report by Liefke et al. (98) demonstrates that the histone demethylase KDM5A/RBP2 is a key component of the Notch CSL repressor complex. Recent studies demonstrate that RBP2 is upregulated in gastrointestinal NETs and in liver metastases from primary NET tumors, suggesting that RBP2 may be actively repressing canonical Notch activity (102) or perhaps remodeling chromatin in these tumors, resulting in aberrant expression of RBP2-regulated genes. Putative inhibitors of RBP2 demethylase activity and/or steric inhibitors to block protein–protein interaction may demonstrate efficacy for this tumor type. Notch receptor, ligand, coactivator, and corepressor function have not been fully analyzed across the spectrum NETs and the complete profile of Notch components present in these diverse tumors may begin to explain the wide variation in clinical responses observed with NETs.

### PI3K/Akt

Genome-wide studies in combination with older immunohistochemical studies have identified components of the PI3K/AKT pathway as dysregulated in NETs (70, 71). Although the PI3K/AKT pathway has been studied for decades in many solid tumors, it has more recently been in the spotlight with respect to GEP-NETs. The PI3K/AKT pathway integrates a multitude of extracellular signals and transmits these signals downstream *via* mTOR. PI3K/AKT/mTOR is the central regulator of a plethora of downstream events, including cell proliferation, apoptosis, cell survival, differentiation, angiogenesis, and cell migration. PI3K can be activated at the cell surface by tyrosine kinase receptors, G-coupled receptors or mutant RAS, and is antagonized by PTEN. Robbins and Hague have conducted a thorough review of PI3K/AKT in GEP-NETs (103), and a few points warrant reemphasis. Pancreatic NETs have a decrease in PTEN expression in 50% of patients, and a downregulation of TSC2 in 35% of patients, both of which correlate with poor survival (71). Small intestinal NETs also have dysregulated PI3K/AKT/mTOR pathway, but exhibit amplification of AKT in 33% of patients (44). This suggests that while there is clear evidence that this pathway is dysregulated in both pancreatic and intestinal NETs, the underlying molecular mechanisms are potentially different, and may explain the varied responses to targeted therapy in clinical trials.

## Conclusion

Gastrointestinal NETs are a very heterogeneous group of tumors. Targeted therapies are available to treat these tumors but despite the many clinical approaches to NETs, and beyond surgery for localized disease, there is little consensus on first line therapy. Results of ongoing clinical trials will better inform patient management with respect to selection, timing, duration, and combination of available therapies, and immunotherapy holds great promise for NETs and other cancers. Furthermore, genetics-based approaches may hold the key toward precision therapies and future investigations into novel pathways may help define driver mutations present in the different subtypes of NETs. Future clinical utilization of gene panels, methylation screening tools and other molecular biomarker approaches in addition to classical neuroendocrine markers, will facilitate treatment and improve outcomes of this disease.

## Author Contributions

JC wrote and edited the entire manuscript.

## Conflict of Interest Statement

The author declares that the research was conducted in the absence of any commercial or financial relationships that could be construed as a potential conflict of interest.
